# Supplementary material for: Chromothripsis during telomere crisis is independent of NHEJ, and consistent with a replicative origin
Source: Genome Res. 2019 May;29(5):737–49. doi: 10.1101/gr.240705.118 (PMC6499312; doi:10.1101/gr.240705.118)
Supplement: Supplemental Material [file supp_gr.240705.118_Supplemental_file_1.zip › contigs/annotated_contigs/DB79/contig.2.DB79_length_414_mean_cov_7.97101449275.docx]

**DB79_length_414_mean_cov_7.97101449275**

```
GAACAGGGTTTCAGCCATCATTCCCTCAGGTATGATGGCGCCGTTATATCTTTTTATCCAGTATACCTGGGTTCAGCTGTTTGCACCTC
 >chr9:93722674-93722996 - E=2e-170
CAAACATCCAATCACTGTTTTCACATTTTTGAGTTTTATGGAATGTGGACAAACTGGAGATCTTTTGTGCTCAGAAAACATTTCAAATT

TCTGCACATCACCTGGCCCTGTGTTTATGTGGGGCTTTGCTCTGTCACATTCTAGAAAGCTCTGAGCCCCTGTGTCAGTTGCCGGCCGA

CCTGACTCATGGTCCTGCTCCTCCTCACAGCAGGGTGTGGACTCAACAGACC|CAG|GTGTCCCCACACTCAGGACTCATGTTGGAGCC
                                                     >chr9:95926797-95926892 + E=1e-45
CTGGGCCCTTACGGTGGGGCAGGAAGACGCCAGCCCCGGTGTGGGCAGTTGGCAGAAAAG
```
